# Chest radiograph reading panel performance in a Bangladesh pneumococcal vaccine effectiveness study

**DOI:** 10.1136/bmjresp-2018-000393

**Published:** 2019-04-15

**Authors:** Eric D McCollum, Salahuddin Ahmed, Nabidul H Chowdhury, Syed J R Rizvi, Ahad M Khan, Arun D Roy, Abu AM Hanif, Farhan Pervaiz, ASM Nawshad U Ahmed, Ehteshamul H Farrukee, Mahmuda Monowara, Mohammad M Hossain, Fatema Doza, Bidoura Tanim, Farzana Alam, Nicole Simmons, Megan E Reller, Meagan Harrison, Holly B Schuh, Abdul Quaiyum, Samir K Saha, Nazma Begum, Mathuram Santosham, Lawrence H Moulton, William Checkley, Abdullah H Baqui

**Affiliations:** 1 Eudowood Division of Pediatric Respiratory Sciences, Department of Pediatrics, School of Medicine, Johns Hopkins University, Baltimore, Maryland, USA; 2 Global Program in Respiratory Sciences, Department of Pediatrics, Johns Hopkins University, Baltimore, Maryland, USA; 3 Health Systems, Department of International Health, Johns Hopkins Hospital and Health System, Baltimore, Maryland, USA; 4 Johns Hopkins University – Bangladesh, Dhaka, Bangladesh; 5 Program in Global Disease Epidemiology and Control, Department of International Health, Bloomberg School of Public Health, Johns Hopkins University, Baltimore, Maryland, USA; 6 Division of Pulmonary and Critical Care, Department of Medicine, School of Medicine, Johns Hopkins University, Baltimore, Maryland, USA; 7 Center for Global Non-Communicable Disease Research and Training, School of Medicine, Johns Hopkins University, Baltimore, Maryland, USA; 8 Department of Pediatrics, Dhaka Shishu Hospital, Dhaka, Bangladesh; 9 Child Health Research Foundation, Dhaka, Bangladesh; 10 Department of Radiology and Imaging, LabAid Cardiac Hospital, Dhaka, Bangladesh; 11 Department of Radiology and Imaging, Dhaka Shishu Hospital, Dhaka, Bangladesh; 12 Department of Radiology and Imaging, National Institute of Cardiovascular Diseases, Dhaka, Bangladesh; 13 Department of Radiology and Imaging, National Institute of Ophthalmology, Dhaka, Bangladesh; 14 Department of Radiology and Imaging, Bangabandhu Sheikh Mujib Medical University, Dhaka, Bangladesh; 15 Division of Infectious Diseases, Department of Medicine, Duke University School of Medicine, Durham, North Carolina, USA; 16 Duke Hubert-Yeargan Center for Global Health, Durham, North Carolina, USA; 17 Duke Global Health Institute, Durham, North Carolina, USA; 18 Maternal and Child Health Division, International Centre for Diarrhoeal Disease Research, Bangladesh (icddr,b), Dhaka, Bangladesh

**Keywords:** Asia, developing countries, respiratory tract diseases, child, infant, pneumococcal vaccines

## Abstract

**Introduction:**

To evaluate WHO chest radiograph interpretation processes during a pneumococcal vaccine effectiveness study of children aged 3–35 months with suspected pneumonia in Sylhet, Bangladesh.

**Methods:**

Eight physicians masked to all data were standardised to WHO methodology and interpreted chest radiographs between 2015 and 2017. Each radiograph was randomly assigned to two primary readers. If the primary readers were discordant for image interpretability or the presence or absence of primary endpoint pneumonia (PEP), then another randomly selected, masked reader adjudicated the image (arbitrator). If the arbitrator disagreed with both primary readers, or concluded no PEP, then a masked expert reader finalised the interpretation. The expert reader also conducted blinded quality control (QC) for 20% of randomly selected images. We evaluated agreement between primary readers and between the expert QC reading and the final panel interpretation using per cent agreement, unadjusted Cohen’s kappa, and a prevalence and bias-adjusted kappa.

**Results:**

Among 9723 images, the panel classified 21.3% as PEP, 77.6% no PEP and 1.1% uninterpretable. Two primary readers agreed on interpretability for 98% of images (kappa, 0.25; prevalence and bias-adjusted kappa, 0.97). Among interpretable radiographs, primary readers agreed on the presence or absence of PEP in 79% of images (kappa, 0.35; adjusted kappa, 0.57). Expert QC readings agreed with final panel conclusions on the presence or absence of PEP for 92.9% of 1652 interpretable images (kappa, 0.75; adjusted kappa, 0.85).

**Conclusion:**

Primary reader performance and QC results suggest the panel effectively applied the WHO chest radiograph criteria for pneumonia.

**Key messages**

- The World Health Organization working group for Chest Radiography in Epidemiological Studies (WHO CRES) recommends that studies using WHO chest radiograph acquisition and interpretation methodologies report on the processes used and the associated performance of these processes.
- This study's results demonstrate that high quality chest radiograph images were obtained and that the radiographic reading panel effectively applied the WHO methodology.
- This report serves as a potential model for sharing the performance of the WHO chest radiograph interpretation processes and justifies the use of these interpretations for future analyses.

## Introduction

Lower respiratory infections are a leading cause of death in children <5 years old worldwide, and the bacterium *Streptococcus pneumoniae* is responsible for the majority of these deaths.^1^ In 2015 alone, *S. pneumoniae* lower respiratory infections, or pneumococcal pneumonia, were estimated to cause nearly 400 000 deaths in children globally.^1^ According to the most recent Global Burden of Disease study, the south Asian country of Bangladesh has the seventh highest global burden of under-five deaths from lower respiratory tract infections, and about 40% of these estimated 21 000 deaths are attributed to pneumococcal infections.^1^

The introduction of pneumococcal conjugate vaccine (PCV) has been highly effective in reducing vaccine serotype invasive pneumococcal disease in low- and middle-income countries.^2^ Judging the effectiveness of PCV against pneumococcal pneumonia poses unique challenges, however, given pneumococcal pneumonia lacks a diagnostic reference standard.^3 4^ In the absence of a diagnostic reference standard for bacterial pneumonia diagnosis, the WHO developed, and recently updated, a methodology for interpreting chest radiographs in epidemiological studies.^5–7^ This methodology was conceived to improve the diagnostic specificity for likely bacterial pneumonia and the reproducibility of chest radiograph interpretations among children, both of which aide in the generalisability of radiographical interpretations across epidemiological studies of different paediatric populations. This methodology is not intended for clinical application. PCV efficacy trials from The Gambia and South Africa used the WHO methodology for interpreting chest radiographs and reported 37% (95% CI, 27% to 45%) and 20% (95% CI, 2% to 35%) reductions in the incidence of radiographic pneumonia among vaccine recipients compared with placebo.^8 9^ These landmark studies established the WHO methodology as a valid endpoint for future vaccine and epidemiological studies.

In March 2015, PCV10 (Synflorix, GlaxoSmithKline) was introduced into the routine immunisation programme of Bangladesh. We designed a large-scale study in Sylhet district in northeast Bangladesh to evaluate PCV10 effectiveness against multiple endpoints among vaccine-eligible children, including WHO-defined radiographic pneumonia.^10^ As a part of the PCV10 effectiveness study, we describe in this report how we implemented the WHO methodology and also evaluated the performance of the panel of readers trained to interpret chest radiographs.

## Methods

### Study setting

**Figure 1:**
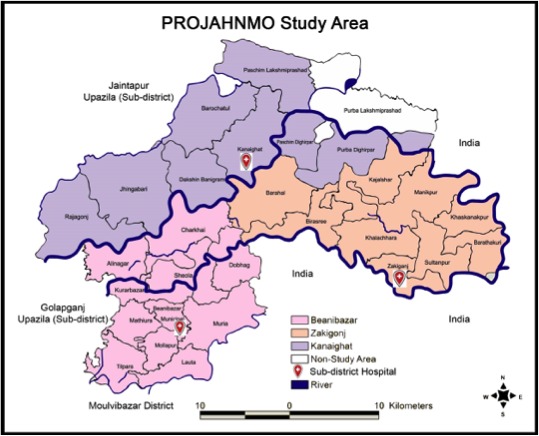
Projahnmo study area in Sylhet, Bangladesh.

The study was conducted at the Projahnmo study group research site between May 2015 and September 2017 in three subdistricts (Zakiganj, Kanaighat and Beanibazar) in the Sylhet district of rural Bangladesh (figure 1). The Projahnmo study group is a consortium that includes Johns Hopkins University, the Government of Bangladesh’s Ministry of Health and Family Welfare (MOHFW), a number of Bangladeshi non-governmental organisations: Shimantik, Child Health Research Foundation and the International Centre for Diarrhoeal Disease Research, Bangladesh. Throughout this study, we performed community-based household surveillance of an estimated 90 000 children <5 years old.^10^ We also conducted facility-based surveillance within outpatient primary care paediatric clinics located at MOHFW subdistrict health complexes in Zakiganj, Kanaighat and Beanibazar during the entire study period. Each health complex serves the population of the surveillance area, and in addition to primary care services, also includes limited inpatient care. Starting December 2016, in an effort to optimise case acquisition, we added the central MOHFW referral hospital Osmani to the study’s surveillance network. At each of the MOHFW clinics, we implemented paediatric computed radiology (CR); at Osmani, we partnered with a private radiology group that used CR.

### Chest radiograph eligibility

Study physicians staffed each MOHFW clinic and were trained to clinically evaluate children for chest radiograph eligibility according to a standardised study protocol. Children aged 3–35 months living in the study’s surveillance area who presented to a study hospital with a history or observation of cough, or a history or observation of difficult breathing, along with signs consistent with possible pneumonia received written informed consent and had chest radiograph imaging. Signs of possible pneumonia included a respiratory rate >50 breaths/min for children aged 3–11 months and >40 breaths/min for those aged 12–35 months, lower chest wall indrawing, persistent nasal flaring, head nodding or tracheal tugging, grunting, stridor, crackles or wheeze on chest auscultation, or wheezing audible without chest auscultation. Starting from October 2015, eligible children also had their oxygen saturation measured by a Rad5 Masimo (Irvine, CA, USA) pulse oximeter with a LCNS Y-1 wrap probe.

### Chest radiograph image acquisition procedures

Children received supine antero-posterior chest radiographical imaging at the time of enrolment by certified radiology technicians who underwent a 1-day protocol and equipment training. We obtained analogue chest radiograph images using portable POLYMOBIL Plus (Siemens, Erlangen, Germany) units and digitised them with CR Fujifilm cassette readers (Tokyo, Japan). The radiology technicians utilised the CR cassette reader to review and upload images to a secure server for archiving and remote access.

### Chest radiograph image acquisition quality control measures

We introduced multiple quality control (QC) measures to optimise chest radiograph images. First, in consultation with a paediatric radiologist, radiology technicians were trained to follow a standardised protocol for obtaining chest radiograph images. Second, during imaging, children were secured in cushioned restraints to minimise movement artefact. Third, at each clinic study, physicians were trained to assess image quality in real time, which provided an opportunity for feedback to technicians on image post-processing and immobilisation of the child. Fourth, each week a random sample of images were shared via a secure server with the expert reader (EDM) for further quality assessment. Any quality issues were flagged and corrective feedback was provided to the sites. Our goal was for <5% of images to be uninterpretable. Fifth, throughout the study, the vendor was available to provide preventative annual servicing, performance assessments and maintenance of radiology equipment.

### WHO chest radiography: interpretation definitions

In this study, we adhered to the WHO methodology for interpreting chest radiographs. See table 1 for these definitions, adapted from Cherian *et al* and Mahomed *et al*.^5 7^

**Table 1:** Definitions of WHO chest radiograph findings (adapted from Cherian *et al* and Mahomed *et al*)^5 7^

Finding		Definition
Quality	Interpretable	Image is sufficiently interpretable for determining the presence or absence of alveolar consolidation
	Uninterpretable	Image is insufficiently interpretable for determining the presence or absence of alveolar consolidation
Classification	Alveolar consolidation	Presence of a dense or fluffy opacity occupying a portion* or whole of a lobe or of the entire lung, which may or may not include air bronchograms and may or may not produce a silhouette sign†
	Other infiltrate	Linear or patchy densities not meeting alveolar consolidation criteria that may also be configured in a lacy pattern in one or both lungs, usually featuring peribronchial thickening or multiple areas of atelectasis
	Pleural effusion	Fluid in the lateral pleural space between the lung and chest wall, not including fluid in the horizontal or oblique fissures
Conclusion	PEP	Presence of alveolar consolidation or pleural effusion that is associated with any type of consolidation (alveolar or other infiltrate)
	Other infiltrate	Presence of other infiltrate not associated with a pleural effusion
	No PEP or other infiltrate	Absence of alveolar consolidation, other infiltrate or pleural effusion

*Defined as a consolidation that has its smallest diameter greater than or the same size as one posterior rib and its adjacent rib space at the same level as the consolidation.

†Defined as the loss of an anatomical border adjacent to any consolidation.

PEP, primary endpoint pneumonia.

### WHO chest radiography: training

Following WHO recommendations, we established a reading panel to interpret chest radiograph images. Eight Bangladeshi readers based in the capital Dhaka comprised the panel and included six radiologists and two paediatricians. In April 2015, each panel member was trained and standardised to the WHO chest radiograph interpretation definitions for 2 days by an international WHO-certified trainer. In November 2015, May and November 2016, and May 2017, in consultation with the WHO-certified trainer, the panel’s expert reader (EDM) conducted twice annual re-standardisation trainings of panel participants to the WHO methodology.

### WHO chest radiography: interpretation procedures

This study had two interpretation schemas: method 1, which included one arbitration level and an expert level, and method 2, which included two arbitration levels and an expert level. All readers were masked to clinical data and other panel member image interpretations. All images were assigned to readers automatically using programmed software. Readers were required to interpret images within 72 hours of receipt, at which time the image was automatically reassigned to another reader. The study was originally designed to use method 1 only; however, method 2 was instituted beginning in November 2016 in an effort to optimise identification of primary endpoint pneumonia (PEP) cases due to slower than expected enrolment. Method 2 was not started in response to any specific reading panel performance issues. After reaching a final classification, the chest radiograph image results were shared with the study physicians and incorporated into patient management. A documented chest radiograph result was also provided to parents at the follow-up household visit.

### Chest radiograph interpretation: method 1

In method 1, two panellists were randomly assigned to be the first two readers (primary readers) for each chest radiograph image. If the two primary readers agreed, the image was interpretable and met criteria for the presence or absence of WHO PEP, then the interpretation was considered final. If primary reader interpretations did not agree, then the image was shared with a randomly selected third reader, the first arbitrator, who was chosen from among the remaining six panellists. The first arbitrator was masked to the notion that they were adjudicating a discordantly interpreted image. If the first arbitrator’s classification agreed with either of the primary readers for the presence or absence of WHO PEP, then the first arbitrator’s interpretation was considered final. However, if the first arbitrator’s interpretation did not agree with either of the first two interpretations, the image was next shared with the expert reader (EDM) and the expert reader’s classification was final.

### Chest radiograph interpretation: method 2

In method 2—from November 2016—all procedures of method 1 were followed except the expert reader also served as a second arbitrator for images interpreted by the first arbitrator as no PEP. In order to harmonise the interpretation methodology for the entire study period, the second arbitrator also retrospectively reviewed all images assessed prior to November 2016 that were similarly interpreted by the first arbitrator as no PEP. The results of method 2 represent the combination of the real time and retrospectively arbitrated reads completed by the expert reader.

### Chest radiograph interpretation QC measures

The expert reader (EDM) was additionally responsible for QC of the reading panel. In addition to facilitating twice annual refresher trainings, supervision included monitoring individual reader performance throughout the study and providing monthly individualised feedback to reading panel members. The expert reader also interpreted a 20% random sample of images each month in which the expert was masked to the panel’s final classification. The panel’s overall performance was then evaluated using the expert’s interpretation as the reference, and this feedback was shared with the reading panel at twice annual refresher trainings. Panel reads that included a second arbitrator or expert interpretation were excluded from this assessment. Lastly, in order to monitor individual reader interpretation reproducibility, each month all readers were randomly reassigned 10% of images they interpreted the prior month.

### Ethics

The Institutional Review Boards of the Johns Hopkins Bloomberg School of Public Health, Johns Hopkins School of Medicine, Bangladesh Institute of Child Health and the Ethical Review Committee of the International Centre for Diarrhoeal Diseases Research, Bangladesh approved the study protocol.

### Statistical analysis

We calculated primary reader agreement after categorising primary reader classifications into binary positive or negative groups for a chest radiograph interpretation or image feature. For interobserver and intraobserver primary reader agreement for the presence or absence of WHO PEP, we included all chest radiographs classified by both primary readers as interpretable. We chose not to adjudicate for WHO-classified other infiltrate in this study based on two factors. First, prior studies were unable to successfully train reading panels to interpret images for WHO-defined ‘other infiltrate’ with high interobserver and intraobserver agreement.^5 11^ Second, our planned analyses for PCV effectiveness were based on PEP only. As in previously designed studies,^12^ if any reader classified the chest radiograph to have WHO other infiltrate, this designation was included in the image’s final panel classification. We used an unadjusted Cohen’s kappa statistic and a kappa statistic adjusted for prevalence and reader bias (prevalence-adjusted, bias-adjusted kappa) to estimate reader agreement not expected by chance.^13^

### Patient and public involvement

Patients and the public in Sylhet, Bangladesh were involved and made aware of the development, design, recruitment and conduct of the study through local community sensitisation meetings held prior to and during the study. Chest radiograph results were disseminated to individual study participants and the overall study results were shared with the public through ongoing community meetings held by the Projahnmo study group consortium in Sylhet, Bangladesh.

## Results

**Figure 2:**
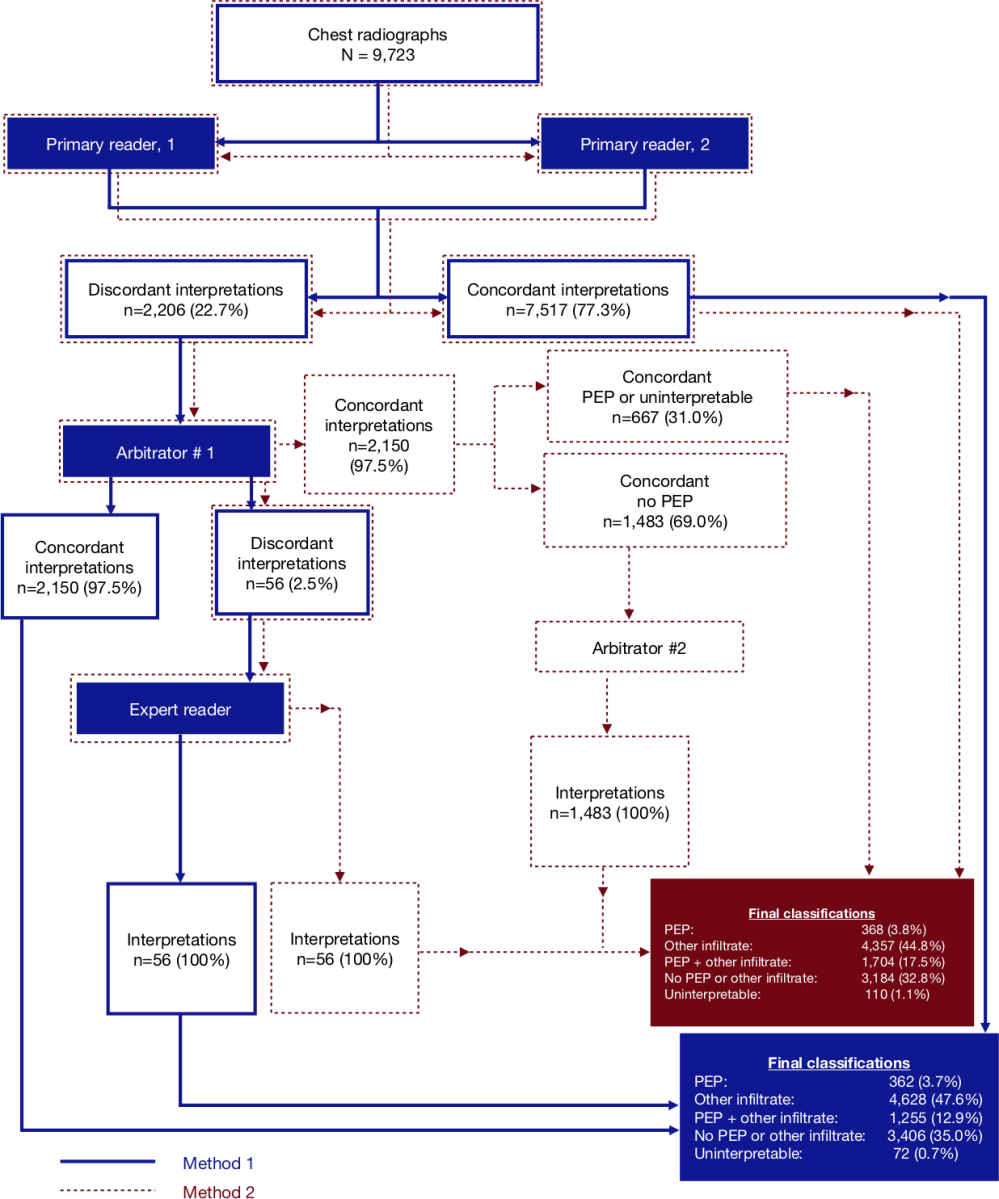
Chest radiograph interpretation schema. Concordance indicates agreement on image interpretability and PEP. PEP, primary endpoint pneumonia.

The overall chest radiograph panel reading process for methods 1 and 2 are depicted in figure 2. From among 9723 total chest radiograph images interpreted using method 1, the panel’s classifications when *excluding* second arbitrator interpretations, the panel interpreted 362 (3.7%) as PEP, 4628 (47.6%) as other infiltrate, 1255 (12.9%) as both PEP and other infiltrate, 3406 (35.0%) as neither, and 72 (0.7%) as uninterpretable. Using method 2, the panel’s classifications when *including* second arbitrator interpretations, the panel classified 368 (3.8%) as PEP, 4357 (44.8%) as other infiltrate, 1704 (17.5%) as both PEP and other infiltrate, 3184 (32.8%) as neither, and 110 (1.1%) as uninterpretable. Method 2 classified an additional 455 images as PEP. The difference between the proportion of PEP cases identified from the 9723 chest radiographs using method 1 versus method 2 was statistically significant with an absolute difference of 4.7% (16.6% vs 21.3%, respectively; p value<0.0001).

**Figure 3:**
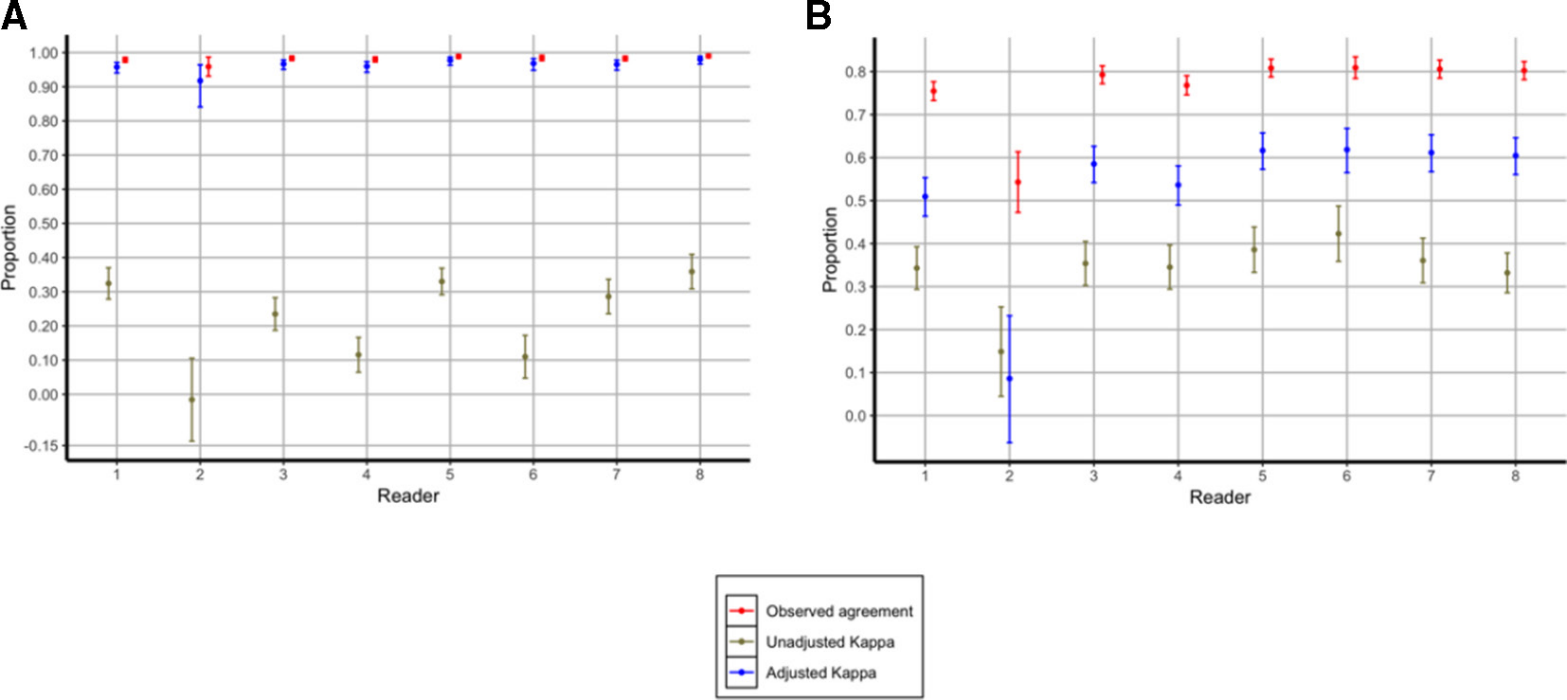
(A) Interobserver agreement for interpretable versus uninterpretable chest radiographs among the eight individual primary readers. (B) Interobserver agreement for PEP versus no PEP chest radiographs among the eight individual primary readers. PEP, primary endpoint pneumonia.

We report the summary primary reader interobserver agreement results in table 2, the individual primary reader interobserver agreement results for image interpretability in figure 3 and the individual primary reader interobserver agreement results for the presence or absence of PEP also in figure 3. The overall primary reader interobserver agreement level for image interpretability from the 9723 images was 98% (unadjusted kappa, 0.25; adjusted kappa, 0.97). From among the 9533 images classified as interpretable by either of the primary readers, interobserver agreement for the presence or absence of PEP was 79% (unadjusted kappa, 0.35; adjusted kappa, 0.57).

**Table 2:** Interobserver agreement at the primary reader level

Characteristic		Frequency of characteristic, n/N (%)*	Overall observed agreement, (%)†	Unadjusted kappa	95% CI	Adjusted kappa‡
Uninterpretable§		28/9723 (0.3)	0.98	0.25	0.23 to 0.27	0.97
	Rotated	2/21 (9.5)	0.86	0.49	0.07 to 0.91	0.71
	Blurry	7/21 (33.3)	0.76	0.52	0.09 to 0.95	0.52
	Over penetrated	0/21 (0)	0.95	0.00	0 to 0	0.90
	Under penetrated	3/21 (14.3)	0.90	0.69	0.26 to 1.12	0.81
	Clipped image	5/21 (23.8)	0.95	0.88	0.45 to 1.3	0.90
Any PEP¶		959/9533 (10.1)	0.79	0.35	0.33 to 0.37	0.57
	Air bronchogram**	14/692 (2.0)	0.87	0.18	0.10 to 0.25	0.75
	Silhouette sign**	360/692 (52.0)	0.72	0.39	0.31 to 0.46	0.45
	Size criteria**	361/692 (52.2)	0.65	0.16	0.09 to 0.24	0.29
	Pleural effusion	45/959 (4.7)	1.00	1.00	0.94 to 1.06	1.00
	Aged 3–11 months	492/4990 (9.9)	0.79	0.36	0.33 to 0.38	0.58
	Aged 12–23 months	311/3016 (10.3)	0.78	0.34	0.30 to 0.37	0.55
	Aged 24–35 months	156/1527 (10.2)	0.79	0.35	0.30 to 0.40	0.57
Right-sided PEP only		480/9533 (5.0)	0.83	0.28	0.26 to 0.30	0.66
Left-sided pleural pneumonia only		120/9533 (1.3)	0.94	0.27	0.25 to 0.29	0.88
Bilateral PEP (both)		58/9533 (0.6)	0.96	0.21	0.19 to 0.23	0.92
Bilateral PEP (any)		269/9533 (2.8)	0.81	0.12	0.10 to 0.14	0.62

*Both primary readers agreed to the presence of characteristic. Discordant interpretations were assumed to not have the characteristic.

†Both primary readers agreed to either the presence or absence of the characteristic.

‡Prevalence-adjusted, bias-adjusted kappa statistic.

§7 Uninterpretable images are missing data on image characteristics.

¶190 of 9723 images were classified as by either primary reader as uninterpretable and were excluded.

**267 of the 959 images classified by primary readers as PEP are missing data on image features.

PEP, primary endpoint pneumonia.

Compared with assessing images for interpretability, we found wider performance variability between individual primary readers for the presence or absence of PEP. Specifically, individual primary reader interobserver agreement levels for interpretability ranged from 96% (reader 2) to 99% (readers 5 and 8), unadjusted kappa values from 0.02 (reader 2) to 0.36 (reader 8) and adjusted kappa values from 0.92 (reader 2) to 0.98 (readers 5 and 8). Interobserver agreement levels for the presence or absence of PEP ranged from 54% (reader 2) to 81% (readers 5, 6 and 7), unadjusted kappa values from 0.15 (reader 2) to 0.42 (reader 6) and adjusted kappa values from 0.09 (reader 2) to 0.62 (readers 5 and 6).

**Figure 4:**
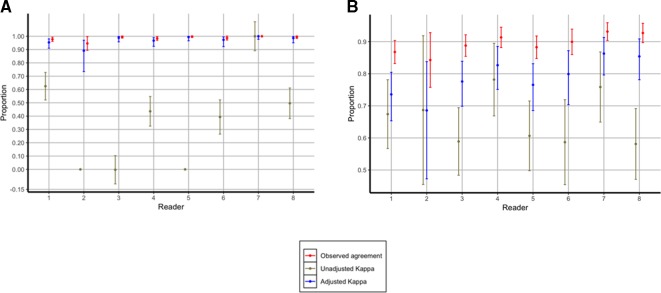
(A) Intraobserver agreement for interpretable versus uninterpretable chest radiographs among the eight individual primary readers. (B) Intraobserver agreement for PEP versus no PEP chest radiographs among the eight individual primary readers. PEP, primary endpoint pneumonia.

To evaluate the reproducibility of image interpretation by individual readers, we reassigned 10% of images to readers for reinterpretation. Readers were masked to the fact that they were reinterpreting an image for the second time. When considering all readers together, intraobserver agreement for all readers was 99.4% (2219/2232) for image interpretability (unadjusted kappa, 0.50; adjusted kappa, 0.98) and 85.1% (1868/2194) for the presence or absence of PEP from among interpretable images (unadjusted kappa, 0.68; adjusted kappa, 0.80). Performance variation between individual readers ranged from 95% (reader 2) to 100% (readers 5 and 7) for agreement on image interpretability, unadjusted kappa values ranged from 0 (readers 2, 3 and 5) to 1.0 (reader 7), and adjusted kappa values from 0.89 (reader 2) to 1.0 (reader 7) (figure 4). Percentage agreement for the presence or absence of PEP ranged from 84% (reader 2) to 93% (readers 7 and 8), unadjusted kappa values from 0.58 (reader 8) to 0.78 (reader 4) and adjusted kappa values from 0.59 (reader 2) to 0.86 (reader 7) (figure 4). Reader 2 was removed from the reading panel from January 2016.

The study’s expert reader interpreted a random subset of 1673 (17.2%) images for QC, none of which were previously interpreted by the expert reader, for QC and determined 1652 (98.7%) of them to be interpretable. Considering the expert reader’s classification as the reference standard, the study’s reading panel achieved a sensitivity of 77.3% (232/300) and specificity of 96.3% (1303/1352) from among the 1652 interpretable images (table 3). In addition, this study’s reading panel identified 82.5% (232/281) and 95.0% (1303/1371) of true positives and true negatives, as classified by the expert reader, using WHO interpretation procedures. When restricting to interpretable images, the panel agreed with the expert’s classification for the presence or absence of PEP on 92.9% of the 1652 images (unadjusted kappa, 0.75; adjusted kappa 0.85).

**Table 3:** Chest radiograph panel performance compared with expert reference interpretations

N=1652*		Expert		
		PEP	No PEP	Total
Panel	PEP	232	49	281
	No PEP	68	1303	1371
	Total	300	1352	1652
Sensitivity		232/300 (77.3%)		
Specificity		1303/1352 (96.3%)		
Positive predictive value		232/281 (82.5%)		
Negative predictive value		1303/1371 (95.0%)		
Per cent agreement, % (n/N)		92.9% (1535/1652)		
Unadjusted kappa		0.75		
Adjusted kappa†		0.85		

*Twenty-one uninterpretable chest radiographs excluded.

†Prevalence-adjusted, bias-adjusted kappa statistic.

PEP, primary endpoint pneumonia.

## Discussion

We report the performance of a reading panel applying the WHO chest radiograph interpretation methodology to 9723 images obtained from rural Bangladeshi children aged 3–35 months with possible community-acquired pneumonia. Our overall results demonstrate high imaging quality, effective application of the WHO methodology by the reading panel, and that WHO PEP is common among rural Bangladeshi children with signs and symptoms of acute respiratory illness.

In addition to raw percentage agreement, in this study we analysed interobserver and intraobserver agreement using two additional approaches, the Cohen’s kappa statistic and the prevalence and bias-adjusted kappa. In most cases, we observed differences between these two estimates. The Cohen’s kappa statistic estimates the percentage of agreement not attributable to chance.^14^ The magnitude of the Cohen’s kappa statistic can be influenced by two factors, the prevalence of the condition of interest (PEP) and observer bias (primary readers).^13^ If prevalence is low, then the probability that two readers will agree simply by chance is higher, and this lowers the kappa’s magnitude.^15^ While in our study the proportion of radiographs with PEP was frequent from an epidemiological standpoint, as we discuss below, prevalence was infrequent from the perspective of the kappa statistic. In statistics, bias is the systematic tendency to overestimate or underestimate the true prevalence.^15^ With respect to kappa, where the true prevalence is unknown, bias may be estimated as the difference in the overall prevalence estimated by two individuals whose ratings are being compared.^13 15^ If the difference in prevalence distribution between two readers is low, then bias is low.^15^ If the difference is high, then bias is high.^15^ Relative to having high bias, low bias lowers Cohen’s kappa.^15^ In our study, bias is likely negligible because our eight different primary readers were randomly assigned to the roles of the two primary readers, and the prevalence estimates for the two raters in the calculation are thus identical.

As name suggests, the prevalence and bias-adjusted kappa statistic is an adaption of the Cohen’s kappa statistic that accounts for the effects of both prevalence and bias.^13^ This adjusted kappa should not be interpreted in isolation, but instead by whether and how much it may differ in magnitude from the Cohen’s kappa.^15^ In this study, we frequently observed that the prevalence and bias-adjusted kappa was higher than the Cohen’s kappa. This difference in magnitude implies that effects from having a low prevalence—from a kappa perspective—and low reader bias are likely present. The prevalence and bias-adjusted kappa is also a tool that facilitates comparisons between studies with different underlying disease prevalence and/or reader tendencies.

Based on raw percentage agreement, Cohen’s kappa statistic, and the prevalence and bias-adjusted kappa, the performance of this study’s reading panel compares favourably with other studies applying the WHO methodology. In the Pneumonia Aetiology Research for Child Health (PERCH), a case–control child pneumonia aetiology study from seven low-income settings, primary readers agreed on the presence or absence of PEP in 77.8% of 3497 interpretable images (unadjusted Cohen’s kappa, 0.50; prevalence and bias-adjusted kappa, 0.56).^11^ In comparison, primary readers in our study achieved 79.0% interobserver agreement (unadjusted kappa, 0.35; adjusted kappa, 0.58). As a measure of interpretation reproducibility, PERCH readers achieved 91% intraobserver agreement (unadjusted kappa, 0.82) for the presence or absence of PEP.^11^ Comparatively, our readers achieved 85.1% intraobserver agreement (unadjusted kappa, 0.68). In a seminal study that reported the interpretation performance of a WHO working group against reference chest radiograph images interpreted according to WHO methodology, the working group achieved 84% sensitivity, 89% specificity, and an unadjusted Cohen’s kappa of 0.65 for clinicians and 0.73 for radiologists detecting PEP.^5^ The expert reader in our study is a member of the global WHO working group for Chest Radiography in Epidemiological Studies.^7^ When considering the expert reader interpretation as the reference standard, our panel achieved a sensitivity of 77%, specificity of 96% and an unadjusted Cohen’s kappa of 0.75. Taken together, these results support the notion that our panel effectively applied the WHO interpretation methodology.

Notably, we observed that the proportion of images classified as PEP increased by 28.1% after adding a second arbitrator to our interpretation schema, and this may have important implications on our PCV effectiveness analyses. Other studies have also demonstrated that the number of arbitrators used to interpret chest radiographs, as well as the types of images obtained, can influence final imaging conclusions. In PERCH, for example, a schema with two arbitrators, compared with one arbitrator, changed the final reading panel conclusion in 27.5% of 4172 images.^11^ Given these findings, we plan to use radiographical conclusions applying both methods, method 1 (one arbitrator) and method 2 (two arbitrators), in our case–control and incident trend PCV effectiveness analyses.

We found that PEP was common—from an epidemiological perspective—among our cohort of predominantly ambulatory Bangladeshi children aged 3–35 months with clinically suspected pneumonia, regardless of the methodology applied (16.6%, method 1; 21.3%, method 2). PERCH, a primarily hospital-based study, reported 27% of 3587 interpretable chest radiographs had PEP.^16^ PERCH also included two sites from Bangladesh with a mix of ambulatory and hospitalised children. The authors reported that about 19% of children from the urban site in Dhaka and 17% of children from the rural site in Matlab had PEP, consistent with our findings from Sylhet. A recent systematic review that included 15 studies from low-income to high-income settings outside of the USA reported a prevalence of 37% (95% CI, 26% to 50%) for radiographic pneumonia among children with an acute respiratory illness.^17^ The four studies including children <5 years old from Asia highlight the range of radiographic pneumonia prevalence within the region. The authors reported a prevalence of 63% from 541 Chinese children, 15% from 1782 Pakistani children, 29% from 199 Philippine children and 7% from 1396 Thai children. Our findings suggest that radiographic PEP among children aged 3–35 months in rural Bangladesh is an important condition.

Limitations of our study include the use of a single frontal chest radiograph rather than both frontal and lateral chest radiographs and the absence of serial imaging. Our approach was carefully considered and based on three principles. First, the WHO methodology does not recommend lateral chest radiographs or serial imaging and use of either would be inconsistent with WHO methodology. Second, evidence supporting the use of lateral imaging among children is mixed. One randomised clinical trial from 570 children seeking care at an emergency department in the USA found no improvement in the sensitivity and specificity of clinician-identified radiographic alveolar consolidation after the addition of a lateral chest radiograph, compared with a frontal chest radiograph alone.^18^ However, a non-randomised retrospective study based on radiologist interpretations found conflicting results.^19^ In this study, the authors reported that the addition of a lateral chest radiograph improved the identification of non-lobar consolidations by 15% compared with frontal imaging alone. Further high-quality research assessing the potential added value of lateral chest radiographs, particularly in low-income settings, is needed. Third, although serial chest radiographs may improve diagnostic sensitivity, it increases ionising radiation exposure and this study’s ethical review boards would not approve a protocol with serial imaging.

In sum, this analysis demonstrates that we have rigorously and effectively applied the WHO methodology for interpreting chest radiographs among a large cohort of children aged 3–35 months in rural Bangladesh. This study provides justification for use of these interpretations in planned case–control and incident trend PCV-effectiveness evaluations in addition to other planned epidemiological analyses.
